# Prognostic Factors of Functional Recovery from Left Hemispheric Stroke

**DOI:** 10.1155/2018/4708230

**Published:** 2018-05-02

**Authors:** Siriphan Kongsawasdi, Jakkrit Klaphajone, Kanokwan Watcharasaksilp, Pakorn Wivatvongvana

**Affiliations:** ^1^Clinical Epidemiology Program, Faculty of Medicine, Chiang Mai University, Chiang Mai, Thailand; ^2^Department of Rehabilitation Medicine, Faculty of Medicine, Chiang Mai University, Chiang Mai, Thailand; ^3^Department of Internal Medicine, Faculty of Medicine, Chiang Mai University, Chiang Mai, Thailand

## Abstract

Although lateralization of the brain affects some specialized cortical functions, there are still limited data to address its influence on clinically important outcomes. This study aimed to reveal the prognostic variables that relate to functional recovery in stroke patients with a left-sided hemispheric lesion during 6 months of follow-up. Data from 167 left-sided and 183 right-sided hemispheric strokes were reviewed retrospectively. Outcomes in this study included walking capacity and functional recovery, assessed by the modified Rankin Scale (mRS). In order to obtain independent predictive variables, this study used the step-backward method of multivariable regression analysis of parameters. The final model demonstrated that motor function of the hemiparetic leg was the strongest independent predictor for both walking ability and functional recovery (risk ratio (RR) of 2.41, 95% CI: 1.61–3.60, and *p* < 0.001 and RR of 1.83, 95% CI: 1.03–3.26, and *p* = 0.04, resp.). Therefore, lateralization did not seem to be involved. Understanding predictable variables that are associated with recovery can guide the rehabilitation team in setting priority and appropriate treatment for stroke patients.

## 1. Introduction

The role of lateralization in the functional outcome of the brain from stroke is not well established. Studies are limited on how the side of the brain on which the lesion appears affects the rate and amount of stroke recovery. Hemispheric lesion may demonstrate differences in some cortical functions, as an individual with left-sided hemispheric stroke (LHS) usually has impaired language comprehension and expression [1–4]. Hemispatial or unilateral neglect, which is characterized by reduced attention or spatial awareness of the body and environment on the hemiplegic side, occurs more often and seriously with a right-sided hemispheric stroke (RHS) [1, 5]. Despite unilateral neglect and aphasia being the most common consequences of neuropsychological deficit after stroke [6], their roles in predicting functional outcome are still inconclusive and have not been reported adequately. Limited studies more likely reveal the relationship of unilateral neglect on functional outcome than a left-sided lesion with aphasic problems [5, 7, 8]. Few studies have reported the functional outcome from side of hemisphere involvement, and they have been inconclusive [1, 3, 5, 6, 9–11]. Differences in the results may be related to heterogeneity of the population, methods, timing of measurements, and variable outcomes [9, 12]. Some studies found that stroke patients with a right-sided hemispheric lesion had a poorer outcome than those with a left-sided one [13–16], whereas others found that those with a left-sided lesion had a poorer outcome [1, 12].

A large prospective acute stroke trial [1] found that hemispheric lateralization was not an independent predictor of functional outcome, as measured by the modified Rankin Scale and NIHSS score. Fink et al. [1] and Woo et al. previously [17] found that if NIHSS is used as a functional outcome, perhaps the NIHSS score is biased in itself. A greater score indicated that severe impairment tended to relate to comprehension deficit of individuals with LHS, that is, language, speech, dysphagia, and facial palsy, rather than impairment following an RHS, that is, inattention. Roles of lateralization on functional recovery are still of interest for rehabilitation professionals, especially in some developing countries with limited resources. Therefore, identifying the key predictor of the optimum functional outcome is essential, since strategic plans could be guided for the rehabilitation decision during the subacute period, for example, as to which patients are suitable for supported home discharge planning. The aim of this study was to explore possible prognostic variables of functional recovery in stroke patients with left-sided hemispheric involvement, which presumably is the dominant side, during 6 months of follow-up.

## 2. Method

A single dataset of ischemic strokes was obtained from the Stroke Unit of the Tertiary University Hospital, Chiang Mai University, Thailand, between January 2010 and March 2015. The inclusion criteria of this study were (1) adults aged above 18 years and diagnosed as having had their first-ever ischemic stroke, (2) no previous disability, and (3) availability of well-documented neurological records at admission and a six-month follow-up.

### 2.1. Clinical Variables and Measurement of Outcome

A retrospective review of medical records, including baseline characteristics, stroke risk factors, comorbidity, and related stroke variables such as motor assessment, stroke consequences, and problems and complications from other causes that might affect recovery, were recorded during hospitalization. All of the stroke-related variables were assessed in the first week after stroke and during six months after onset period. Motor performance of upper and lower extremities was assessed by manual muscle testing. Perceptual deficit was assessed by observing patient responses. The deficits were defined as patients failing to respond to stimuli provoked on the hemiplegic side or being unable to perceive their body parts via a conventional standardized subtest (i.e., the line bisection test or copy drawing test) [18]. Apraxia is the inability to perform skilled and purposeful motor tasks despite having the physical ability to do. This phenomenon was assessed by observing patient behavior such as inability to perform purposeful movements, errors when asked to demonstrate how to use an object or common instruments, and problems imitating abstract and symbolic gestures (e.g., wave goodbye and salute like a soldier) [19]. Speech and communication impairment were assessed by difficulty in fluency, comprehension, and repetition. Reassessment of these variables was revised at follow-up within six months after onset. The outcomes in this study were the ability to walk and functional recovery assessed by a modified Rankin Scale (mRS), which indicated improvement during 6 months of follow-up. “Walking capacity” referred to the ability to walk on a level surface for at least 10 meters and allowing the use of a gait aid or orthotic device, and “functional recovery” was defined according to a mRS score of 1 (no significant disability) to 3 (moderate disability: requiring some help but able to walk without assistance). The mRS is a clinician-reported measure of global disability which has been applied widely for evaluating recovery from stroke, particularly regarding physical disability and the need for assistance. It was reported as having a strong relationship with other clinical measurements of stroke severity and sensitivity in order to identify mild and moderate disability in acute stroke management [18]. Clinical variables and outcomes were assessed by certified physicians, and all recordings were approved and countersigned by expert academic physicians. The Ethics Committee of the Faculty of Medicine, Chiang Mai University, Thailand, approved the study protocol on 24 July 2015 (Research ID: NONE 2558-03123).

### 2.2. Statistical Analysis

Baseline characteristics, stroke risk factors, motor assessment, and complications during hospitalization were compared between patients with LHS and RHS. Fisher's exact test was used for categorical variables and Student's *t*-test performed for normally distributed continuous variables, with a significance level of *p* < 0.05. Univariable analysis of clinical variables was performed, with dependent variables being the outcomes of walking capacity and functional recovery, and all independent variables, having a dichotomous scale (0 = no; 1 = yes), were clinically meaningful variables. There was no confounding variable in this prognostic study design. Statistically significant variables (*p* < 0.01) from the univariable analysis were submitted to the multivariable analysis model. The step-backward method of multivariable risk regression analysis was used, with the generalized linear model, in order to derive the final independent predictive variables that were shown as the risk ratio (RR) and 95% confident interval (CI), in which *p* < 0.05 indicated statistical significance. All statistical analyses were performed utilizing STATA version 12 Software.

## 3. Results

**Figure 1:**
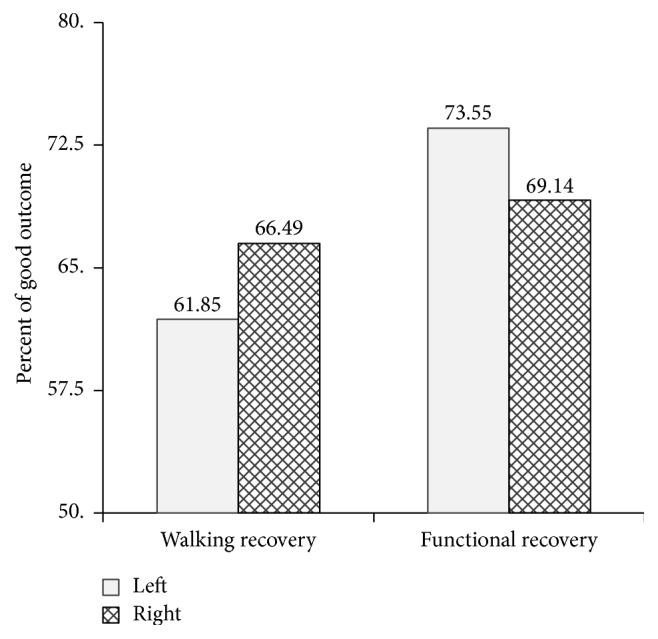
Proportion of patients with a left- or right-sided hemispheric lesion who achieved walking and functional recovery.

Of the 350 ischemic stroke patients who met the inclusion criteria, 167 were individuals with LHS and 183 had RHS. There was no significant difference in the distribution frequency of ischemic type between the two groups (*p* = 0.58). The main etiology of a large vascular lesion was atherosclerosis on either the left side (72.46%) or right side (71.58%).  Table 1 demonstrates no significant difference in the distribution of baseline demographics (i.e., age, gender, ischemic stroke subtype, and blood pressure on admission) or clinical data (i.e., prior transient ischemic attack (TIA), health status, and comorbidity prior to stroke) among individuals with LHS and RHS, except for the initial Glasgow Coma Score (GCS) and diabetes mellitus (DM), in which a left-sided hemispheric lesion showed a significantly lower GCS and greater number of DM cases than the right-sided one (*p* < 0.05, Fisher's exact test). The proportion of patients who achieved walking and functional (mRS score of 1–3) recovery (Figure 1) was not statistically different between those with left- or right-sided hemispheric lesions. A proportion of 62 and 66 percent of individuals with LHS and RHS, respectively, could walk independently on a level surface at 6 months' follow-up. Regarding functional outcome, 74 and 69 percent of individuals with LHS and RHS, respectively, accounted for functional recovery (mRS score 1–3). No significant difference between the two groups was found in either walking or functional recovery (*p* > 0.05, Fisher's exact test).  Table 2 demonstrates and compares the motor ability and poststroke complications between individuals with LHS and RHS. Motor performance of the hemiparetic arm and leg was not significantly different between the two groups, as measured by manual muscle testing (MMT). Almost all poststroke related complications showed no significance between the two groups except for communication and perception problems. LHS patients had the greater number of communication problems with 23.1% and 27.8% having motor or sensory aphasia and global aphasia, respectively. 3.2% and 4.3% of right-sided hemispheric stroke patients had motor or sensory aphasia and global aphasia (*p* < 0.001), respectively. Nevertheless, the individuals with LHS had more perceptual disturbance and inattention (unilateral neglect) than those with RHS (17.3 versus 4.05%, *p* < 0.001).

### 3.1. Prognostic Factors for the Postacute Stage after Stroke

This study focused on prognostic variables of the left hemisphere, which was assumed to be the dominant side of the brain. Univariable analysis of prognostic factors related to functional and walking recovery as shown in Table 3. Aphasia and motor function of the hemiparetic arm and leg were found to be associated strongly with either functional or walking capacity during 6 months' follow-up (*p* < 0.01). Despite inattention being found commonly with a right-sided hemispheric lesion, a left-sided one was found to relate with functional recovery. Poststroke complications were related to walking ability. Nevertheless, the final stepwise multivariate model demonstrated that only motor function of the leg was the strongest independent predictor for both functional and walking recovery in this setting, with a risk ratio (RR) of 2.41, 95% confidence interval [CI] of 1.61–3.60, and *p* < 0.001 and RR of 1.83, 95% CI of 1.03–3.26, and *p* = 0.04, respectively (Table 4).

## 4. Discussion

### 4.1. Functional Outcome between Left- and Right-Sided Lesions

This study found an insignificant trend in the proportion of patients who achieved a clinical outcome in either functional recovery (mRS score of 1–3) or walking capacity between left- and right-sided lesions, despite them being well matched for demographic data and major variables associated with the outcome (Table 1). However, GCS on admissions with a left-sided lesion had a significantly lower score than those with a right-sided one (*p* < 0.001), which was similar to a previous study [10].

Evidence predicting stroke outcome by side of hemisphere is still inconclusive from previous studies. Considering terms of anatomical and hemodynamic aspects, left-sided hemispheric infarction, both atherosclerotic and cardioembolic stroke with underlying hypertension, has been revealed as being more frequent and more severe and having worse outcome than right-sided hemispheric infarction [10, 19]. This could be due to higher intima-media thickness and mean flow velocity of circulation in the left carotid artery, which reflects greater hemodynamic stress in the left cerebrovascular system and finally enhances atherogenesis, as evidenced from hemodynamic study [20]. Left-sided hemispheric stroke tended to have poorer outcomes, due to a higher incidence of large left-sided hemispheric vessels and middle cerebral artery distribution [21]. In contrast, Goto et al. [11] found that, in most cases of middle cerebral artery, locomotion outcome in right-sided hemispheric infarction was poorer than that in a left-sided lesion, except in a case of large infarction.

### 4.2. Independent Predictors of Functional Outcome in LHS

The results from this study demonstrated the effect of exposure on the outcome of interest by using the step-backward method of multivariable risk regression model, risk ratio (RR). This finding revealed that the functional outcome in LHS was not affected by side of brain involvement but only by motor function of the leg, which was the strongest independent predictor for both functional recovery and walking recovery. Univariable analysis demonstrated that aphasia, inattention, and motor function of the arm and leg were associated in the subacute period with walking ability, while aphasia, medical complications, and motor function of the arm and leg were associated with functional recovery. Nevertheless, by recruiting all significant variables from univariable analysis, motor function of the leg was the only independent factor in either walking recovery or functional recovery in the final stepwise multivariate model.

The communication problem, aphasia, is a major consequence of a left-sided hemispheric lesion [1, 2]. With the current use of high gamma electrocorticography, the inferior frontal gyrus of the dominant left hemisphere is demonstrated as being one of the most important brain regions for language processing [4]. Aphasia and dysarthria have been reported as independent factors associated with functional status [21], increased length of hospital stay, and complications during acute stroke admission [20, 21]. Poststroke survivors with aphasia demonstrated negative impact on health-related quality of life (HRQoL) in a systematic review, with emotional distress and limited social relationships [22], increased rate of mortality [23], and poor gait quality [22]. In recent studies, Kim reported that aphasia and dysarthria affect the recovery of activities in daily living, quality of life, cognitive function, motor power, and ambulation status [24]. Laska et al. [23] demonstrated retrospectively from the Virtual International Stroke Trials Archive that aphasia at baseline and at 3 months and persistent dysarthria at 3 months were associated significantly with a poorly modified Rankin Scale in a large cohort (*n* = 8,904). In this study, aphasia was found to relate negatively to the outcomes in univariable analysis but not in the final model of multivariate analysis. Although aphasia is thought to be a negative factor in outcomes, the findings of this study, and a previous one by these authors [25], indicate that only motor function was the most predictive factor of functional outcomes in either ambulation (RR, 2.41; 95% CI: 1.61–3.60) or overall functional ability (RR, 1.83; 95% CI: 1.03–3.26) when adjusting for other possible prognostic variables in a multivariate analysis. A systematic review [26] and recent article in 2015 [27] confirmed that the initial grade of paresis was the most important predictor with respect to early prognosis of motor recovery. The greatest overall improvement in motor functions was evidenced within the first month after stroke, with some degree of motor recovery continuing for up to 6 months, especially in groups of initially severe patients [25]. These findings supported the importance of rehabilitative training strategies toward improving motor performance in the subacute period and referral to appropriate local community resources in order to enhance the mechanism of recovery after stroke [28–30].

### 4.3. Limitations

The small number of subjects was a limitation of this study which could not stratify data according to subtypes of ischemic stroke, which might affect the outcome; that is, lacunar infarct and a small vessel occlusion should reach a better outcome than main territory infarct [11, 14]. In addition, the retrospective nature of this study omitted some possible clinical variables in the regression analysis. Other variables from a multidimensional perspective such as background of the patients prior to stroke, that is, level of fitness, side preference or related factors after stroke, motivation, attitude, and compliance to the rehabilitation training program, might have contributed to the outcome but could not be included as variables in this analysis. These aspects may have limited interpretation ability, and a larger, prospective study is needed for verification.

## 5. Conclusion

This study demonstrated that motor function of the leg was the strongest independent predictor of walking and functional ability in left-sided ischemic stroke patients. Findings from this study indicate that the determinants of functional outcome may be actual impairments rather than hemispheric involvement. Although this study might not shed new light on predicting functional recovery in the stroke population, its research methodology could be conducted practically on a routine basis and transferred into established guidelines worthy of assisting rehabilitation teams in individualizing appropriate strategic plans for stroke patients.

## Figures and Tables

**Table 1:** Demographic data, premorbid health status, and stroke-related characteristics of individuals with left- and right-sided hemispheric lesion at baseline (*n* = 350).

Characteristics	Left hemisphere (*n* = 167)		Right hemisphere (*n* = 183)		*p* value
Gender					1.00
Male	89	53.29	97	53.00	
Female	78	46.71	86	46.99	
Subtype					0.58
Large artery atherosclerosis	121	72.46	131	71.58	
Cardioembolism	4	2.39	2	1.09	
Small-vessel occlusion	42	25.15	50	27.32	
Age, mean (±SD)	63.71 (13.41)		63.98 (12.76)		0.85
Blood pressure, mean (±SD)					
Admission SBP	155.99 (30.06)		152.4 (28.37)		0.25
Admission DBP	87.8 (20.34)		86.6 (17.53)		0.57
Glasgow Coma Score	12.8 (2.34)		14.0 (1.84)		<0.001^*∗∗*^
Prior TIA	14	8.09	13	7.03	0.69
Atrial fibrillation	44	25.43	62	33.51	0.24
Smoking	46	26.59	59	31.89	0.30
Alcohol consumption	25	14.45	34	18.38	0.39
Comorbidity					
Hypertension	121	72.45	123	67.21	0.29
Dyslipidemia	98	58.68	97	53.00	0.30
Diabetes mellitus	47	28.14	30	16.39	<0.01^*∗*^
Other comorbidities					0.20
(1) Comorbidity	53	31.73	53	28.96	
(2) More comorbidities	41	24.55	60	32.78	

TIA, transient ischemic attack; SBP, systolic blood pressure; DBP, diastolic blood pressure; ^*∗*^*p* < 0.01 and ^*∗∗*^*p* < 0.001.

**Table 2:** Comparison of six-month follow-up motor assessment and poststroke complications between left- and right-sided hemispheric lesions.

Characteristics	Left hemisphere (*n* = 167)		Right hemisphere (*n* = 183)	
	*N*	%	*N*	%
Follow-up motor assessment				
MMT arm				
Grade 0-1	53	31.74	63	34.42
Grade 2-3	62	37.12	61	33.33
More than 3+	52	31.14	59	32.24
MMT leg				
Grade 0-1	52	31.14	60	32.79
Grade 2-3	62	37.12	61	33.33
More than 3+	53	31.74	63	34.43
Stroke-related complications				
Aphasia^*∗∗∗*^				
Dysarthria	46	26.59	93	50.27
Motor/sensory aphasia	40	23.12	6	3.24
Global aphasia	48	27.75	8	4.32
Inattention^*∗∗∗*^	7	4.05	32	17.30
Dysphagia	17	9.83	10	5.41
Apraxia	3	1.73	2	1.08
Depression	9	5.20	13	7.03
Other complications				
(1) Complication	42	24.42	36	19.57
(2) More complications	17	9.88	22	11.96

^*∗∗∗*^ *p* < 0.001.

**Table 3:** Independent predictors of walking and functional recovery from left-sided hemispheric stroke from a univariable analysis with the generalized linear model.

Predictors	RR	95% CI	*p* value
*Walking recovery*			
Aphasia	1.86	1.23–2.19	<0.001^*∗∗∗*^
Inattention	2.37	1.02–5.49	0.04^*∗*^
Motor: arm	2.52	1.73–3.67	<0.001^*∗∗∗*^
Motor: leg	2.65	1.83–3.84	<0.001^*∗∗∗*^
*Functional recovery*			
Aphasia	1.65	1.16–2.36	<0.01^*∗∗*^
Poststroke complications	1.73	1.78–2.54	<0.01^*∗∗*^
Motor: arm	2.23	1.62–3.74	<0.001^*∗∗∗*^
Motor: leg	2.47	1.76–3.28	<0.001^*∗∗∗*^

RR, risk ratio; CI, confidence interval; ^*∗*^*p* < 0.05; ^*∗∗*^*p* < 0.01; ^*∗∗∗*^*p* < 0.001.

**Table 4:** Independent predictors of walking and functional recovery of left-sided hemispheric stroke within 6 months after stroke using multivariable analysis with the generalized linear model.

Predictors	RR	95% CI	*p* value
*Walking capacity*			
Motor: leg	2.41	1.61–3.60	<0.001^*∗∗*^
*Functional recovery*			
Motor: leg	1.83	1.03–3.26	0.04^*∗*^

RR, risk ratio; CI, confidence interval; ^*∗*^*p* < 0.05; ^*∗∗*^*p* < 0.001.
